# Cardiovascular health in Brazilian state capitals ^1^

**DOI:** 10.1590/1518-8345.1327.2843

**Published:** 2017-10-19

**Authors:** Fernanda Penido Matozinhos, Mariana Santos Felisbino-Mendes, Crizian Saar Gomes, Ann Kristine Jansen, Ísis Eloah Machado, Francisco Carlos Félix Lana, Deborah Carvalho Malta, Gustavo Velaquez-Melendez

**Affiliations:** 2 PhD, Adjunct Professor, Escola de Enfermagem, Universidade Federal de Minas Gerais, Belo Horizonte, MG, Brazil.; 3 Doctoral student, Escola de Enfermagem, Universidade Federal de Minas Gerais, Belo Horizonte, MG, Brazil. Scholarship holder from Coordenação de Aperfeiçoamento de Pessoal de Nível Superior (CAPES), Brazil.; 4 PhD, Associated Professor, Escola de Enfermagem, Universidade Federal de Minas Gerais, Belo Horizonte, MG, Brazil.; 5 PhD, Researcher, Departamento de Vigilância de Doenças e Agravos Não Transmissíveis e Promoção da Saúde, Secretaria de Vigilância em Saúde, Ministério da Saúde, Brasília, DF, Brazil.; 6 PhD, Full Professor, Escola de Enfermagem, Universidade Federal de Minas Gerais, Belo Horizonte, MG, Brazil.

**Keywords:** Health, Cardiovascular System, Epidemiologic Factors, Health Surveys, Health Promotion

## Abstract

**Objective:**

to estimate the prevalence of ideal cardiovascular health indicators in the Brazilian population, according to gender, age, education and region of residence.

**Method:**

cross-sectional study that used data from 41,134 participants of the Surveillance System of Risk and Protective Factors for Chronic Diseases by Telephone Survey (Vigitel). The ideal cardiovascular health assessment considers four behavioral factors: not smoking; body mass index less than 25 kg/m^2^; practicing physical activity, eating fruits and vegetables five or more times per day; and two clinical factors (no diagnosis of diabetes or hypertension). The sum of factors at ideal levels results in a score ranging from zero (worse cardiovascular health) to six (ideal cardiovascular health).

**Results:**

considering the six factors, only 3.4% of the studied population presented ideal levels of cardiovascular health, with the majority of participants (57.6%) presenting three or four ideal factors. Women had higher prevalence of ideal cardiovascular health (3.8% versus 2.9% for men) (p < 0.0001).

**Conclusion:**

the findings of this study are consistent with the elevated risk of mortality from cardiovascular disease, observed in the Brazilian population. This may contribute to a better understanding of the scenario of cardiovascular health in the urban population of the country.

## Introduction 

The high prevalence of cardiovascular diseases (CVD) is a consequence of changes in the life habits of the population^1^, and in 2010 these diseases were among the top 20 responsible for Disability Adjusted Life Years (DALYs) in Brazil^2^. Inadequate diet, hypertension, alcohol consumption, being overweight and smoking, are, in this order, the five major risk factors for these diseases^2^.

The American Heart Association (AHA) has proposed measures to evaluate the cardiovascular health of populations through the simultaneous presence of seven factors, four behaviors (not smoking, performing regular physical activity, body mass index (BMI) <25 kg/m^2^ and a healthy diet) and three clinical factors (Cholesterol <200 mg/dl, blood pressure <120/80 mmHg and fasting blood glucose <100mg/dl)^3^. Some studies indicate that the presence of six or seven of these factors at ideal levels is associated with a reduction of 70% to 89% in the incidence of cardiovascular diseases, when compared to groups that have none or just one of these factors at ideal levels^3^ ^-^ ^5^.

Epidemiological investigations that allow the evaluation of the cardiovascular health of the population are essential for the direction of public policies that promote healthy life habits^1^. In Brazil, Since 2006, the Surveillance System of Risk and Protective Factors for Chronic Diseases by Telephone Survey (Vigitel) has been monitoring risk factors in the Brazilian population residing in the capitals^6^. In this context, Vigitel has proved to be a potentially suitable database for this evaluation, despite the majority of factors being self-reported.

Thus, the aim of this study was to estimate the prevalence of ideal cardiovascular health indicators among the population, according to gender, age, education and region of residence.

## Method 

The present study examined 2012 data of the *Sistema de Vigilância de Fatores e Risco e Proteção para Doenças Crônicas por Inquérito Telefônico* (VIGITEL) . The Vigitel evaluates, by means of a telephone interview, risk and protective factors for chronic non-communicable diseases, among people aged 18 years and over, resident in the capitals, in the 26 Brazilian states and the Federal District. More detailed information about the Vigitel system is described in a previous publication^6^.

In 2012, in all the 27 cities, 45,448 individuals were interviewed. The study exclusion criteria were: pregnant women (n = 317), those who did not know whether they were pregnant (n = 42), participants who did not present weight or height data and those who did not know whether they had a previous diagnosis of hypertension and/or diabetes (n = 3,955), giving a total of 41,134 individuals.

Cardiovascular health was evaluated as proposed by the American Heart Association (AHA)^3^, with some adaptations. Of the seven indicators recommended, six were evaluated, four behavioral (smoking, body mass index (BMI), physical activity and consumption of fruits and vegetables) and two clinical factors (diabetes and hypertension). Moreover, in this study, the factors were self-reported and there were not data for dyslipidemia. Another adaptation refers to the diet questionnaire, which include only consumption of fruits and vegetables^3^.

The six factors were classified as: ideal (1) and poor (0). The following conditions were considered ideal: not smoking (never smoked); BMI <25 kg/m^2^; performing physical activity (>150 minutes weekly of light or moderate intensity physical activity or >75 minutes weekly of vigorous physical activity in all areas); consumption of fruit, legumes and vegetables (except potatoes, yucca and yam) five or more times per day, for five or more days per week; and no self reported previous medical diagnosis of diabetes and hypertension. Finally, cardiovascular health was evaluated from the sum of these six factors; which ranged from zero (worst cardiovascular health) to six (ideal cardiovascular health). Later, the four behavioral factors were grouped as proposed by the AHA^3^. Thus, individuals could present between zero and four behavioral factors at ideal levels.

Data were analyzed using the survey module of the Statistical Software for Professionals (Stata), version 14, taking into consideration the weights used by the Vigitel, i.e., considering the representativeness of the sample. The analysis calculated the prevalence of each factor individually and together. The prevalence was also calculated according to: gender (male, female), age group (18 to 34, 35 to 54, 55 or more years), education level (0-8, 9-11, 12 or more years of study) and region of residence (Central-West, South, Southeast, Northeast, and North). The statistical differences were evaluated by Pearson’s Chi-squared test (p < 0.05). Finally, associations between the socio-demographic variables (age, education and region of residence) and cardiovascular health for men and women were estimated, using Prevalence Ratio (PR) as a measure of association, with 95% confidence intervals (95% CI), obtained by Poisson regression with robust variance^7^. For this specific analysis, ideal cardiovascular health was considered when five or six factors were at ideal levels and also, when the sum of ideal behaviors (three to four behavioral factors) were at ideal levels^5^.

The consent form was replaced with verbal consent; since it was obtained when telephone contact was made with the interviewee. The Vigitel was approved by the National Research Ethics Committee of the Ministry of Health, with decision No. 749/2006 and registration No. 13,081. No identification data were requested from the respondents, with their anonymity and the confidentiality of the information guaranteed.

## Results

A total of 41,134 individuals were included in this study, with mean age (±SE) of 41 years (±0.15). Gender distribution was even, with 48.4% of the participants being male.

**Figure 1:**
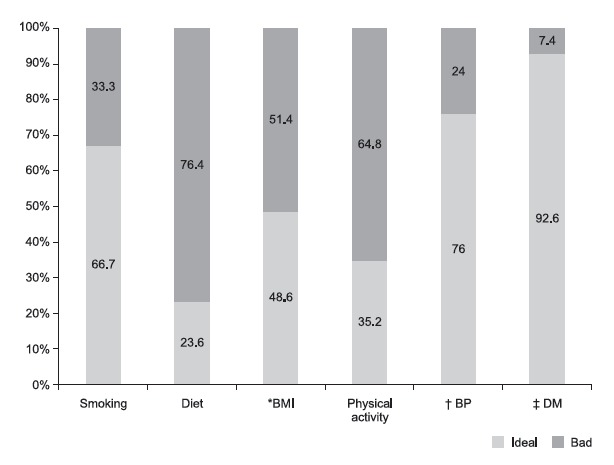
Distribution of cardiovascular health factors in the study population - Surveillance System of Risk and Protective Factors for Chronic Diseases by Telephone Survey. Brazil, 2012

When the individual cardiovascular health factors were analyzed, it was observed that diet presented the lowest level of adequacy, with only 23.6% of the Brazilian population presenting the diet at an ideal level, followed by physical activity (35.2%) and BMI (48.6%) (Figure 1).

* BMI = Body Mass Index; ^†^ BP = Blood Pressure; ^‡^ DM = *Diabetes Mellitus*

When considered individually, women presented better results in some factors, such as: non-smoking (73.2%); normal BMI (52%) and adequate diet (28.8%); conversely, the men presented higher prevalence of ideal levels in: physical activity (42.1%), blood pressure (78.3%) and diabetes (93.3%) (Table 1).

**Table 1:** Distribution of cardiovascular health factors, by gender - Surveillance System of Risk and Protective Factors for Chronic Diseases by Telephone Survey. Brazil, 2012

Factors		Gender			
		Male		Female	P value *
		% (SE)		% (SE)	
Smoking					< 0.001
	Ideal	59.6 (0.8)		73.2 (0.6)	
	Poor	40.4 (0.8)		26.8 (0.6)	
Diet					< 0.001
	Ideal	18.1 (0.6)		28.8 (0.6)	
	Poor	81.9 (0.6)		71.2 (0.6)	
Body Mass Index					< 0.001
	Ideal	45.1 (0.8)		52 (0.6)	
	Poor	54.9 (0.8)		48 (0.6)	
Physical Activity					< 0.001
	Ideal	42.1 (0.8)		28.7 (0.6)	
	Poor	57.9 (0.8)		71.3 (0.6)	
Blood Pressure					< 0.001
	Ideal	78. 3 (0.6)			
	Poor	21.7 (0.6)		26.2 (0.5)	
Diabetes					0.004
	Ideal	93.3 (0.4)		91.9 (0.3)	
	Poor	6.7 (0.4)		8.1 (0.3)	

* Pearson’s Chi-square

**Figure 2:**
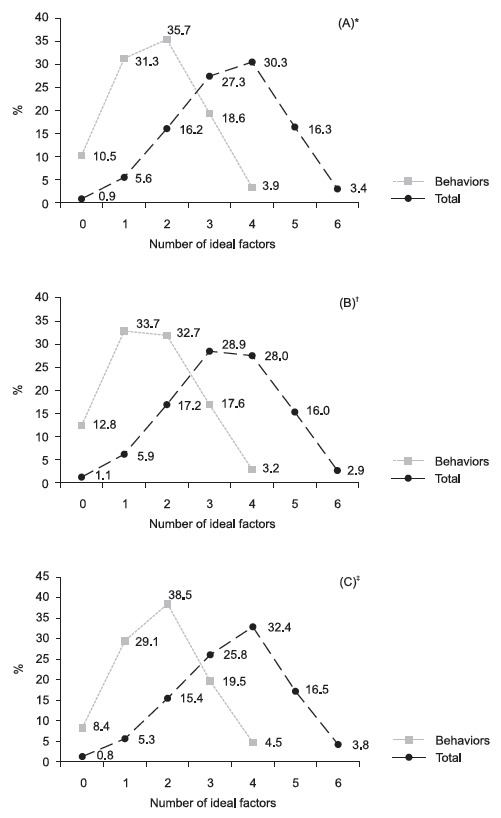
Sum of cardiovascular health indicators and behavioral indicators in the total population and according to gender - Surveillance System of Risk and Protective Factors for Chronic Diseases by Telephone Survey. Brazil, 2012

When analyzing the set of indicators (Figure 2), it was verified that 3.4% of the Brazilian population presented ideal cardiovascular health (all six factors at ideal levels) and, in relation to the sum of the behavioral factors, only 3.9% of the population presented all four factors at ideal levels (Figure 2 - A). Figure 2 shows that 19.7% of the population presented five or six factors at the ideal level and 22.5% three or four behavioral factors at ideal levels.

* A = Total population; ^†^ B = Men; ^‡^ C = Women Differences between male and female - Pearson’s Chi-square p-value < 0.001

In relation to the sum of the six factors, according to gender, the women presented higher prevalence of ideal cardiovascular health (p < 0.0001)(Figure 2 -B and C). The same was observed in the sum of the behaviors (p < 0.0001). Women also presented higher prevalence of five or six factors (20.3%), and of three or four behaviors at ideal levels (24.0%).

When analyzing the prevalence according to sociodemographic characteristics, regardless of gender, individuals with more education, younger and resident in capitals of the Central-West region, presented the highest prevalence of cardiovascular health (five to six factors at ideal levels). Men living in the capitals of the Southern region also presented higher prevalence of cardiovascular health. Similar findings were observed for the prevalence of three or four behaviors at ideal levels (Table 2).

**Table 2:** Prevalence ratio and 95% CI for the number of ideal factors, by gender - Surveillance System for Risk and Protective Factors for Chronic Diseases by Telephone Survey. Brazil, 2012

Sociodemographic Characteristics		3-4 behaviors at ideal levels						5-6 factors at ideal levels				
		Women			Men			Women			Men	
		%*	PR (95% CI)		%*	PR (95% CI)		%*	PR (95% CI)		%*	PR (95% CI)
Education (years)												
	12 or more	35.5	Ref.		29.5	Ref.		32.7	Ref.		27.6	Ref.
	9-11	24.2	0.68 (0.62-0.75)^†^		26.2	0.89 (0.76-1.00)		20.8	0.64 (0.58-0.71)^†^		24.2	0.88 (0.78-0.99)^†^
	0-8	14.0	0.40 (0.35-0.45)^†^		9.2	0.31 (0.26-0.38)^†^		9.1	0.28 (0.23-0.33)^†^		7.4	0.27 (0.21-0.33)^†^
Age group (years)												
	18-34	29.9	Ref.		31.5	Ref.		28.7	Ref.		30.6	Ref.
	35-54	21.3	0.71 (0.65-0.79)^†^		13.5	0.43 (0.38-0.49)^†^		17.9	0.62 (0.56-0.69)^†^		11.0	0.36 (0.31-0.41)^†^
	55 or more	18.7	0.62 (0.56-0.70)^†^		10.9	0.35 (0.29-0.41)^†^		10.2	0.35 (0.31-0.41)^†^		7.4	0.24 (0.19-0.30)^†^
Region of residence												
	Central-West	28.4	Ref.		24.0	Ref		24.7	Ref.		21.5	Ref.
	South	25.5	0.90 (0.80-1.00)^†^		24.4	1.02 (0.80-1.18)		21.4	0.87 (0.76-0.98)^†^		22.6	1.05 (0.90-1.23)
	South East	23.8	0.84 (0.75-0.94)^†^		19.3	0.80 (0.69-0.94)^†^		19.8	0.80 (0.70-0.91)^†^		17.7	0.82 (0.70-0.97)^†^
	Northeast	22.6	0.79 (0.73-0.87)^†^		20.6	0.86 (0.76-0.97)^†^		19.2	0.77 (0.70-0.86)^†^		18.3	0.85 (0.75-0.97)^†^
	North	22.5	0.79 (0.71-0.88)^†^		22.4	0.94 (0.81-1.08)		19.8	0.80 (0.71-0.90)^†^		20.1	0.93 (0.80-1.09)

* Population prevalence; ^†^ p-value < 0.05; PR: Prevalence Ratio; 95% CI: 95% Confidence Interval

Lower levels of education were also associated with lower prevalence of ideal cardiovascular health, regardless of gender (PR = 0.28; 95% CI; 0.23-0.33 for women and PR = 0.27; 95% CI; 0.21-0.33 for men), compared to those with higher level of education. Furthermore, the more advanced age groups were associated with lower prevalence of ideal cardiovascular health in both genders, when compared to the younger age group. In this comparison, individuals aged 35-54 years presented prevalence of five or six factors at ideal levels, about 40% lower for women and 70% lower for men; while for those in the 55 years or more age group this was 65% lower for women and 76% lower for men. In relation to regions of residence, for women, living in capitals of all regions was associated with lower prevalence of ideal cardiovascular health when compared to the Central-West region, been the lowest prevalence in the Northeast and Northern regions (PR = 0.77; 95% CI; 0.70-0.86 and PR = 0.80; 95% CI; 0.71-0.90, respectively). With regard to men, living in the Southeast and Northeast regions was associated with a prevalence of about 15% less of five or six ideal factors, compared with those living in the Central-West. Similar results were found when assessing sociodemographic factors and the sum of the behaviors (three or four behaviors at ideal levels) (Table 2).

## Discussion

This study, conducted with a probabilistic sample of adults from all 27 Brazilian capitals, showed that the population presented low levels of ideal cardiovascular health indicators; better performance was detected among women, younger individuals (with higher educational level) and residents of the capitals of the Central-West region of the country. These results are consistent with the high rates of disability-adjusted life years (DALYs) and with the high risk of mortality due to cardiovascular diseases, observed in the Brazilian population^1^ ^-^ ^2^.

Some studies conducted in cities of the United States, Europe and China, found prevalence values of cardiovascular health at the ideal level lower than those shown in this study (respectively, 1.0%, 1.0% and 0.5%)^8^ ^-^ ^10^, while a systematic review of 50 studies conducted worldwide, showed a large variation in this prevalence, ranging from 0.3% to 15.0%, with the worst outcomes in developing countries (0.3% to 4.0%)^5^.

The prevalence values found in this study should be interpreted with caution. An explanation for the observed differences may have been related to the self-reported diagnosis of diabetes and hypertension, since biochemical and clinical alterations can be present in individuals without the confirmed diagnosis of the disease. This possible underestimation of the presence of diabetes and hypertension is reinforced by the analysis of behavioral factors. It is well known that a sedentary lifestyle, inadequate diet and excessive weight, are risk factors for the two pathologies investigated in this study^1^ ^-^ ^2^ ^,^ ^11^ ^-^ ^12^.

It should be noted that in the above mentioned studies, seven factors were evaluated and not six, as in the present one. An investigation that used a similar strategy and evaluated the same six self-reported indicators, showed that less than one in 10 Canadian adults (9.4%) had ideal cardiovascular health from 2003 to 2011^13^.

When evaluating only the behavioral factors, diet presented the lowest prevalence of adequacy, followed by physical activity, body weight and smoking, corroborating the recent report on the global burden of diseases. This report highlighted inadequate diet as the main risk factor for DALYs^2^, indicating the need to continuously work on this indicator to improve its quality in the population.

In several studies, diet was the worst performing behavioral factor^5^ ^,^ ^8^ ^,^ ^10^ ^,^ ^13^. There are public policies that aim to respond to the scenario found and are consolidated in various strategies, such as the National Policy on Food and Nutrition, and the Food Guide for the Brazilian Population and the National Food and Nutrition Safety System. However, they are still obstacles to promoting healthy eating among the population, such as availability and costs of healthy foods, as well as access to information. A study carried out with data from the Research of Family Budgets (RFB) in Brazil pointed out that a reduction of 1% in the price of fruit and vegetables would lead to an increase of 0.79% in the consumption of these products, mainly in the population with lower purchasing power^14^. The increase in the consumption of these foods is also related to production, storage, local processing of fruits and vegetables, and to the concomitant presence of food education programs that guide the population to consume these foods^11^.

In relation to behavioral factors, physical activity is an important factor for the prevention and control of cardiovascular diseases^1^ ^,^ ^12^. Lack of physical activity and insufficient physical activity were responsible for approximately 3.2 million deaths and 2.8% of DALYs in the global population^12^. In Brazil, the Health Academy program (2011), implemented by the Ministry of Health, aimed, among other aspects, to promote public facilities with infrastructure, equipment and qualified professionals for the orientation of physical activity, in the same way that it promotes health actions, trying to encourage physical activity and improve the health of the population.

With regard to a BMI greater than 25 kg/m^2^, an association with an increased risk of cardiovascular diseases was observed^1^. Excess weight and its associated diseases are a constantly growing problem in Brazil and worldwide. This is a challenge for health managers due to the impact on the quality of life^15^. The double challenge for this intervention requires the improvement of the dietary pattern and the increase in physical activity^11^ ^,^ ^15^.

In turn, smoking is a worrying public health problem, since smoking increases the chances of acquiring cardiovascular diseases^1^ ^,^ ^16^. The high prevalence of the smoking factor at the ideal level was also found in other studies^5^ and results from numerous successful programs in smoking prevention and smoking cessation in the country. The higher prevalence of smoking among men is linked to historical and cultural aspects^17^.Furthermore, higher proportions of tobacco smokers are found among people with lower levels of education^18^.

The higher prevalence of ideal levels of cardiovascular health among women was also found in a Canadian study, in which 12.8% of the female population presented cardiovascular health indicators at ideal levels, compared to only 6.1% of male population^13^. Better cardiovascular health was also found among younger and more educated individuals, consistent with other population-based studies, and this may be related to, for example, increased physical activity in individuals in these groups^6^ ^,^ ^19^.

The risk of diabetes and hypertension increases with age^17^. A population-based cohort study with 2,164 adults, between the ages of 18 and 30 at baseline, showed that after the age of 25, cardiovascular health of 80.3% of the population worsened, throughout the study. The authors also demonstrated that adequate BMI and not smoking were protective factors for not losing the ideal level of cardiovascular health, regardless of gender and ethnicity^20^.

According to studies conducted in high income countries, chronic non-communicable diseases tend to be more frequent in populations with lower levels of education, which has also become a reality in low and middle income countries^21^. For example, individuals with more schooling tend to practice more physical activity, with numerous barriers, including socio-economic aspects, contributing to a sedentary lifestyle, which in turn decreases cardiovascular health^22^. According to data from the 2010 Global Burden of Disease study, physical inactivity and insufficient physical activity were responsible for approximately 3.2 million deaths and 2.8% of the potentially lost years due to premature death adjusted for disability, worldwide^12^. The small number of physically active individuals worldwide is partially due to the lack of guidance and suitable premises for performing physical activities^23^.

Finally, in the present study, residents of capitals of the Central-West and men living in the Southern region had the highest prevalence of ideal cardiovascular health; the worst results were found in the Northern and Northeastern regions. A study published in 2015 on the burden of disease in Brazil according to its regions, showed a greater burden of disease (including chronic diseases) in the North and Northeast of the country; which could be a consequence of worse living conditions and a more restricted access to health services in these regions^24^, corroborating the low prevalence of ideal cardiovascular health found in these regions in this study.

Some limitations should be considered in this study. First, this study used self-reported information (an inherent aspect of telephone surveys), which is easy to perform in large population samples. It should be mentioned that the use of self-reported data has been widely applied in epidemiological studies and is considered an acceptable and valid method^25^. Dyslipidemia was not included in this study because it was not evaluated in the Vigitel questionnaire between 2010 and 2012. A previous study also presented this limitation, which did not prevent the joint evaluation of the indicators^13^. Another limitation refers to the adaptation of the diet behavioral factor, which in this study was obtained from a questionnaire of food consumption more simplified than the one proposed by the AHA^3^. It should also be mentioned that the individuals who reported being ex-smokers were included in the smoking category. Finally, it is emphasized that physical activity was also evaluated by a more simplified questionnaire than the one proposed by the AHA, which may have led to overestimation.

In spite of the potential limitations, there are few population studies with urban representativeness that have estimated the prevalence of ideal cardiovascular health factors in low- and middle-income countries.

## Conclusion

In conclusion, a very small proportion of adults in the Brazilian capitals presented cardiovascular health factors at ideal levels; and there were differences according to gender, age and education level. Despite the fact that women, the youngest, the most educated and the residents of the Central-West region had better cardiovascular health, the prevalence was still considered very low. In general, these results could indicate a negative impact on the DALYs and on the global burden of disease, which leads to the reduction of longevity and the increase of disabilities throughout life.

The findings of this study may contribute to a better understanding of the cardiovascular health scenario in the Brazilian urban population and may also guide the implementation of different prevention approaches, mainly the promotion of both individual and collective health. These findings can also help to create effective public intervention policies and to expand programs aimed at improving the quality of life in cities, with the final aim that individuals achieve ideal levels of the cardiovascular health factors.
